# Initial biological evaluations of ^18^F-KS1, a novel ascorbate derivative to image oxidative stress in cancer

**DOI:** 10.1186/s13550-019-0513-x

**Published:** 2019-05-17

**Authors:** Kiran Kumar Solingapuram Sai, Nagaraju Bashetti, Xiaofei Chen, Skylar Norman, Justin W. Hines, Omsai Meka, J. V. Shanmukha Kumar, Sriram Devanathan, Gagan Deep, Cristina M. Furdui, Akiva Mintz

**Affiliations:** 1 Department of Radiology, Wake Forest School of Medicine, Winston Salem, NC 27157 USA; 2 Department of Chemistry, Koneru Lakshmaiah Education Foundation, Guntur, Andhra Pradesh 522502 India; 3 Department of Internal Medicine, Section on Molecular Medicine, Wake Forest School of Medicine, Winston Salem, NC 27157 USA; 4 Medical Guidance Systems LLC, St. Louis, MO 63108 USA; 5 Department of Cancer Biology, Wake Forest School of Medicine, Winston Salem, NC 27157 USA; 6 Department of Radiology, Columbia University Irving Medical Center, New York, NY 10032 USA

**Keywords:** Positron emission tomography (PET), Prostate cancer, Head and neck squamous cancer, Ascorbate, Biodistribution

## Abstract

**Background:**

Reactive oxygen species (ROS)-induced oxidative stress damages many cellular components such as fatty acids, DNA, and proteins. This damage is implicated in many disease pathologies including cancer and neurodegenerative and cardiovascular diseases. Antioxidants like ascorbate (vitamin C, ascorbic acid) have been shown to protect against the deleterious effects of oxidative stress in patients with cancer. In contrast, other data indicate potential tumor-promoting activity of antioxidants, demonstrating a potential temporal benefit of ROS. However, quantifying real-time tumor ROS is currently not feasible, since there is no way to directly probe global tumor ROS. In order to study this ROS-induced damage and design novel therapeutics to prevent its sequelae, the quantitative nature of positron emission tomography (PET) can be harnessed to measure in vivo concentrations of ROS. Therefore, our goal is to develop a novel translational ascorbate-based probe to image ROS in cancer in vivo using noninvasive PET imaging of tumor tissue. The real-time evaluations of ROS state can prove critical in developing new therapies and stratifying patients to therapies that are affected by tumor ROS.

**Methods:**

We designed, synthesized, and characterized a novel ascorbate derivative (*E*)-5-(2-chloroethylidene)-3-((4-(2-fluoroethoxy)benzyl)oxy)-4-hydroxyfuran-2(5H)-one (KS1). We used KS1 in an in vitro ROS MitoSOX-based assay in two different head and neck squamous cancer cells (HNSCC) that express different ROS levels, with ascorbate as reference standard. We radiolabeled ^18^F-KS1 following ^18^F-based nucleophilic substitution reactions and determined in vitro reactivity and specificity of ^18^F-KS1 in HNSCC and prostate cancer (PCa) cells. MicroPET imaging and standard biodistribution studies of ^18^F-KS1 were performed in mice bearing PCa cells. To further demonstrate specificity, we performed microPET blocking experiments using nonradioactive KS1 as a blocker.

**Results:**

KS1 was synthesized and characterized using ^1^H NMR spectra. MitoSOX assay demonstrated good correlations between increasing concentrations of KS1 and ascorbate and increased reactivity in SCC-61 cells (with high ROS levels) versus rSCC-61cells (with low ROS levels). ^18^F-KS1 was radiolabeled with high radiochemical purity (> 94%) and specific activity (~ 100 GBq/μmol) at end of synthesis (EOS). Cell uptake of ^18^F-KS1 was high in both types of cancer cells, and the uptake was significantly blocked by nonradioactive KS1, and the ROS blocker, superoxide dismutase (SOD) demonstrating specificity. Furthermore, ^18^F-KS1 uptake was increased in PCa cells under hypoxic conditions, which have been shown to generate high ROS. Initial in vivo tumor uptake studies in PCa tumor-bearing mice demonstrated that ^18^F-KS1 specifically bound to tumor, which was significantly blocked (threefold) by pre-injecting unlabeled KS1. Furthermore, biodistribution studies in the same tumor-bearing mice showed high tumor to muscle (target to nontarget) ratios.

**Conclusion:**

This work demonstrates the strong preliminary support of ^18^F-KS1, both in vitro and in vivo for imaging ROS in cancer. If successful, this work will provide a new paradigm to directly probe real-time oxidative stress levels in vivo. Our work could enhance precision medicine approaches to treat cancer, as well as neurodegenerative and cardiovascular diseases affected by ROS.

**Electronic supplementary material:**

The online version of this article (10.1186/s13550-019-0513-x) contains supplementary material, which is available to authorized users.

## Background

Excessive production of reactive oxygen species (ROS) and/or reactive nitrogen species (RNS) through either endogenous or exogenous insults results in oxidative stress [1]. Oxidative stress has been implicated in many disorders such as cancer and neurodegenerative and cardiovascular diseases [2–4]. Several recent findings have demonstrated the role of ROS and induced oxidative stress in cancer initiation, promotion, and progression [1, 5, 6]. ROS as second messengers modulate several transcription factors and signal transduction molecules such as heat shock-inducing factor and nuclear factors in cancer [7, 8]. ROS and RNS actively participate in regulating cell adhesion, redox-mediated amplification of immune response, and programmed cell death [9]. ROS-mediated oxidative stress induces apoptosis both in tumor and healthy cells [10]. ROS plays a critical role in maintaining cellular homeostasis and physiological redox potential, and their activity is believed to be concentration-dependent [11, 12]. However, other contradicting evidence points to the protective effect of ROS on tumors, including evidence that blocking ROS decreases the efficacy of antitumor drugs ranging from common chemotherapies to newer targeted agents. Thus, it is critical to develop translational tools to monitor real-time ROS to better understand their role in oncogenesis as well as to monitor global tumor ROS pre- and post-therapy to inform potential stratification strategies for personalized therapeutic optimization. However, measuring cellular concentrations of ROS is challenging due to their short half-life and high reactivity profile [13]. Multiple analytical approaches, including electron spin resonance (ESR), electron paramagnetic resonance (EPR), enzymatic probes, chemiluminescence, and fluorescence, have been used to detect ROS/RNS [14–18]. However, all these techniques have inherent issues, including poor sensitivity, regional specificity, and selectivity [19–21]. Hence, there is an unmet need for sensitive, reliable, and quantifiable methods of measuring in vivo ROS levels for both research and clinical use. PET is a noninvasive, fully quantitative, and highly sensitive imaging modality that can detect biomarkers in vivo [22].

PET imaging uniquely offers quantitative potential at picomolar concentrations that do not perturb biologic systems, making it an ideal translational modality to monitor real-time ROS in cancer patients. The dihydroethidium (DHE, a red fluorescent dye) family of probes [23] has been tested as potential PET imaging agents to track ROS for neurological studies [24–27]. Other groups have reported on the use of non-DHE-based PET tracers to track ROS for several biomedical uses [13, 28–31]. Despite the ongoing imaging research in developing novel ROS probes, the underlying in vivo mechanisms of ROS alterations in cancer progression, especially with antioxidants like ascorbate, still remain largely unknown [15, 20, 32, 33]. Therefore, a novel probe that is PET compatible and binds to ROS with high specificity and ideally with existing clinical safety records could provide a solid platform to image ROS alterations in a tumor tissue. Ascorbate (vitamin C or ascorbic acid) is a water-soluble antioxidant with a long-standing favorable safety profile [34]. Recently, Carroll et al. have demonstrated sodium ion (Na^+^)-dependent transport mechanisms of ascorbic acid [35, 36] in mice brain using [^11^C]-ascorbic acid and corroborated oxidative sensitivity of ascorbic acid in vivo [37]. The radiolabeling methods employed were slightly cumbersome and with poor reaction yields. Furthermore, the short life (20 min) of the [^11^C] PET isotope limits its translational scope. Thus, the development of a clinically relevant PET radiotracer that can be used in cancer is yet to be developed.

In this study, we have successfully synthesized and characterized a novel ascorbate derivative, (*E*)-5-(2-chloroethylidene)-3-((4-(2-fluoroethoxy)benzyl)oxy)-4-hydroxyfuran-2(5H)-one (KS1), based on the structure-activity relationship (SAR) of a reported ascorbic acid derivative [38]. KS1 was screened for in vitro ROS binding in cancer cell lines and compared with ascorbate binding. Based on initial in vitro ROS specificity, we produced ^18^F-KS1 and evaluated serum stability, in vitro ROS selectivity, and specificity in head and neck squamous cell carcinoma (HNSCC) and prostate cancer (PCa) cell lines. Furthermore, we investigated the tumor imaging properties of ^18^F**-**KS1 using microPET imaging and biodistribution studies in mice implanted with PCa.

## Methods

### Synthesis

**Fig. 1:**
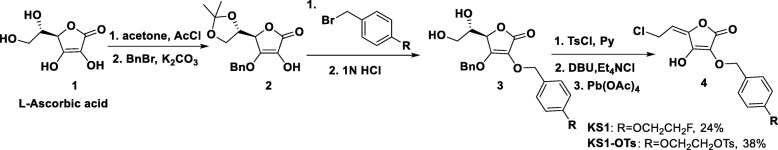
Synthetic scheme for production of KS1

Gazivoda et al. reported that multiple di-aryl-substituted l-ascorbic acid analogs exhibited cytotoxicity against malignant tumor cell lines, including cervical, breast, pancreatic, prostate, and colon carcinoma [38]. The most potent among them, *Z*-2,3-di-*O*-benzyl-6-chloro-4,5-didehydro-l-ascorbic acid, was our design lead for (*E*)-5-(2-chloroethylidene)-3-((4-(2-fluoroethoxy)benzyl)oxy)-4-hydroxyfuran-2(5H)-one (KS1) production scheme. The synthetic scheme (Fig. 1) was derived based on previous reports with slight modifications [38–44]. Briefly, 5^1^, 6^1^-dihydroxyl groups of l-ascorbic acid (1) were protected by acetone/acetyl chloride to obtain a ketal intermediate, 2. The ketal intermediate 2 was then reacted with 4^1^-bromobenzyl-substituted ethoxy alkanes, followed by acid deprotection to give 2^1^-benzyloxy, 5^1^, 6^1^-dihydroxyl alkyl-substituted ascorbic acid, 3. Dehydration and selective 3^1^-debenzylation of the isolated product from the previous step 3 resulted in KS1. The corresponding F-18 radiochemistry precursor unit KS-OTs was prepared using the ethoxy tosyl-substituted alkanes with intermediate 2, followed by deprotection and dehydration steps.

### In vitro testing of KS1

A panel of SCC-61 and rSCC-61 cell lines derived from patients with HNSCC, a genetically matched model of radiation resistance developed by Furdui’s group, was employed [45–49]. The molecular and cellular properties of this model have been well characterized using systems-level analyses and complementary assays. SCC-61 and rSCC-61 cells show differences in (a) response to radiation (SCC61 D0=1.3, rSCC-61 D0=2.0), (b) response to the EGFR targeted inhibitor erlotinib (SCC­61 IC_50_ > 50 μM; rSCC-61 IC_50_ 4.5 μM); (c) cellular phenotype (SCC­61 is mesenchymal while rSCC­61 is epithelial), and importantly (d) ROS levels (SCC-61 has higher intracellular ROS than rSCC-61) [45, 47]. MitoSOX is a mitochondrion-targeted dihydroethidium-based derivative that primarily detects ROS, especially superoxide anion radical, produced within mitochondria [47, 50]. In addition to testing KS1 with the MitoSOX assay, we compared our results with no ligand controls and ascorbate as a reference standard. The two cell lines were treated with KS1 and ascorbate (10.0 μM) and incubated for an hour. MitoSOX (1.0 μM) in phenol red-free DMEM/F12 media was added and incubated for an additional 10 min. The cells were then washed with PBS and imaged in the same media. Ligand specificity is commonly determined by pre-treating the cells with high concentrations of blockers. Superoxide dismutase (SOD), an enzyme that selectively suppresses accumulation of ROS-superoxide peroxide anions, was added as a blocker (1.0 mM) 60 min before the treatment of KS1 [51]. Fluorescence was subsequently measured, and its intensity was quantified in f.u (fluorescence units).

### Radiochemical synthesis of ^18^F-KS1

With both the precursor tosylates and nonradioactive F-19 reference standard in hand, the radiochemical synthesis of ^18^F**-**KS1 was optimized on the TRASIS AIO radiochemistry module (Fig. 3) [52], following the typical ^18^F-F^¯^-based nucleophilic substitution reaction from the corresponding ascorbate tosylate [53]. Briefly, ^18^F-F^¯^ produced from our GE PETtrace cyclotron was azeotropically dried and reacted with corresponding tosylate precursor in DMF at 110 °C for 15 min. Semi-preparative HPLC separation and solid phase C18 sepPak purification and elution with 10% absolute ethanol in saline resulted in ^18^F-KS1 [53–55]. The isolated radioactive product was used for quality control analyses, in vitro cell uptake, and animal studies. The chemical and radiochemical purity and specific activity of the collected radioactive aliquots were determined by HPLC injection on a QC C18 reverse phase column. All radiochemical yields were determined by HPLC collection of ^18^F-KS1, unless stated otherwise. The ex vivo serum stability of ^18^F-KS1 was analyzed in human serum samples, following previously published methods [56–58]. Briefly, radiotracer was added to the human serum sample and incubated at 37 °C. Radioactive serum mixture was injected into the QC-HPLC system at 5 min, 30 min, 1 h, 1.5 h, 2 h, 2.5 h, and 3 h post-radiotracer synthesis.

### Cell uptake studies

ROS efficacy of ^18^F-KS1 was evaluated in HNSCC SCC-61 and rSCC-61cells following published protocols by our group [56, 59, 60]. Fresh solutions of KS1 and SOD (10 μM) were added as ROS blockers to the seeded SCC-61 cells 60 min before radiotracer addition (*n* = 6 per blocker). Additionally, SCC-61 cells were blocked with ascorbate (10 μM) for 60 min before radiotracer addition (*n* = 3). All cells were then treated with ^18^F-KS1 (0.074 GBq/well) and incubated for 60 min (*n* = 6) at 37 °C. All cells (both with and without blockers) were washed three times and lysed with lysate buffer solution (1.0 M NaOH solution). Lysate samples from each well were collected for gamma counting. Similarly, in another experiment, human PCa-PC3 cells were cultured under normoxic (~ 21% O_2_) or hypoxic (1% O_2_) conditions for 48 h. Thereafter, similar to HNSCC cells (detailed above), all assay steps including blocker treatment, radiotracer addition, incubations, washings, and lysis were carried out under normoxic or hypoxic conditions. All hypoxia experiments were performed in a hypoxia chamber (Biospherix X3 Xvivo system). Plates without radioactivity were used as controls. Additional aliquots were taken from each well to measure protein concentration. The counts per minute values of each well were normalized to the amount of radioactivity added to each well and were expressed as percent uptake relative to the control condition. The data were expressed as %ID/mg of protein present in each well.

### In vivo evaluations of ^18^F-KS1

Athymic nude mice (Taconic Farms) were housed in a pathogen-free facility of the Animal Research Program at Wake Forest School of Medicine under a 12:12-h light/dark cycle and fed ad libitum. All animal experiments were conducted under IACUC approved protocols in compliance with the guidelines for the care and use of research animals established by Wake Forest Medical School Animal Studies Committee. PC3 cells (1 × 10^5^ cells suspended in 10 μL Matrigel) were implanted in the left flank of nude mice (25–30 g) as described previously [61–63]. Mice bearing subcutaneous human PCa-PC3 tumors [61–63] were separated into two groups for baseline and blockade studies (*n* = 3/group) and underwent microPET imaging under ~ 1% isoflurane-oxygen anesthesia. Mice were intravenously injected with ~ 3.7 ± 0.30 GBq of ^18^F-KS1 and 45 min later were scanned for 20 min using a TriFoil microPET scanner. KS1 (15 mg/kg) was used as a blocking agent and was injected 45 min before the radiotracer injection. Standard biodistribution studies were conducted in mice bearing PCa tumors to confirm in vivo binding of ^18^F-KS1. Mice were intravenously injected with ^18^F-KS1 (~ 3.7 GBq) and euthanized after 30 min and 60 min of tracer injections (3 mice/time point). Samples of tumor, blood, brain, heart, lung, liver, spleen, pancreas, kidney, muscle, and bone were harvested, weighed, and gamma counted with a standard dilution of the injectate [25, 53, 64]. The percentage of the injected dose per gram of tissue (%ID/g) was calculated.

## Results

### Synthesis

The desired products KS1-OTs and KS1 were synthesized with 38% and 24% chemical yields, respectively (Fig. 1). All intermediates and final compounds were completely characterized using ^1^H NMR and mass spectroscopy. The precursor molecule, (E)-2-(4-(((5-(2-chloroethylidene)-4-hydroxy-2-oxo-2,5-dihydrofuran-3-yl)oxy)methyl)phenoxy)ethyl 4-methylbenzenesulfonate, KS1-OTs was obtained as a white solid, 38% yield and with ^1^H NMR (400 MHz, CDCl_3_): δ 10.15 (s, 1H), 7.82 (d, 2H, *J* = 8.4 *Hz*), 7.38–7.33 (d, 2H, *J* = 8.4 *Hz*), 7.27–7.22 (d, 2H, *J* = 7.8 *Hz*), 6.79–6.77 (d, 2H, *J* = 7.8 *Hz*), 5.46 (t, 1H, *J* = 8.4 *Hz*), 5.12 (s, 2H), 4.39–4.36 (m, 2H), 4.29 (d, 2H, *J* = 8.4 *Hz*), 4.18–4.16 (m, 2H), and 2.46 (s, 3H); MS: 481.96 [M + H]^+^. The nonradioactive standard, (*E*)-5-(2-chloroethylidene)-3-(4-(2-fluoroethoxy)benzyloxy)-4-hydroxyfuran-2(5H)-one, KS1 was isolated as a light brown solid, 24% yield and ^1^H NMR (300 MHz, CDCl_3_): δ 10.12 (s, 1H), 7.32–7.31 (d, 2H, *J* = 2.8 *Hz*), 6.89–6.88 (d, 2H, *J* = 2.8 *Hz*), 5.50–5.46 (t, 1H, *J* = 7.1 *Hz*), 5.19 (s, 2H), 4.86–4.83 (m, 1H), 4.70–4.65 (m, 1H), 4.39–4.37(d, 2H, *J* = 10.4 *Hz*), 4.28–4.27 (m, 1H), and 4.19–4.16 (m, 1H); MS: 329.05 [M + H]^+^.

### In vitro ROS assay

**Fig. 2:**
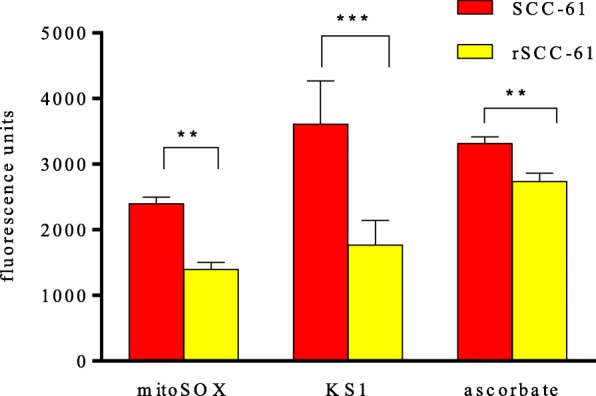
Fluorescence MitoSOX assay of KS1 in two differentially ROS-expressing HNSCC cell lines (SCC-61 and rSCC-61). MitoSOX was the control, and ascorbate was the standard. The data was expressed as measured fluorescence units, with ^**^p ≤ 0.05 and ^***^p ≤ 0.005 considered statistically significant (*n* = 6)

KS1 and ascorbate showed a similar amount of fluorescence (Fig. 2). Both KS1 and ascorbate demonstrated higher fluorescence in the higher ROS-expressing SCC-61 cells compared to the lower ROS expressing-rSCC-61cells. Good correlations were found between increasing concentrations of KS1 (and ascorbate) from 1.0 μM to 100 μM and increased fluorescence. KS1 exhibited ~ 2.2-fold higher differential selectivity between the high- and low-ROS cell lines compared to ascorbate (Fig. 2). In order to further establish the specificity of KS1 for ROS, the same MitoSOX assay was performed by pre-treating the SCC-61 cells with SOD (1.0 mM), an ROS blocker, for 60 min. Fluorescent uptake decreased by ~ 50% demonstrating KS1 specificity.

### Radiolabeling of KS1

**Fig. 3:**
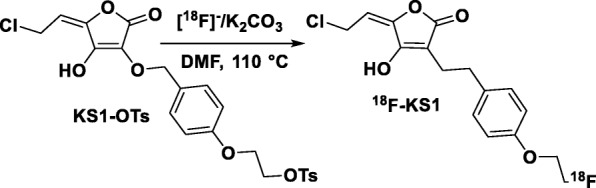
Synthetic scheme of ^18^F-KS1 production

Given the promising preliminary binding and specificity of KS1 (compared to ascorbate), KS1 was radiolabeled with ^18^F^¯^ to obtain ^18^F-KS1 (Fig. 3). Radiochemical synthesis, including [^18^F]F^¯^ transfer, reaction, HPLC purification, and radiotracer formulation, was completed within 65 min (Additional file 1: Figure S1). Injection of ^18^F-KS1 showed a single radioactive peak with minimal UV absorbance, indicating good specific activity. The radioactive peaks were further authenticated by performing a co-injection with their corresponding nonradioactive standard KS1, which displayed similar retention times. ^18^F-KS1 was synthesized with high radiochemical purity (> 94%) and high specific activity ~ 100 ± 10 GBq/μmol (decay corrected to end of synthesis; EOS). ^18^F-KS1 was produced in 15% decay-corrected radiochemical yield (*n* > 10). The ex vivo serum stability of ^18^F-KS1 was studied in a human serum sample at 5 min, 30 min, 1 h, 1.5 h, 2 h, 2.5 h, and 3 h post-radiotracer synthesis. ^18^F-KS1 was ~ 90% intact in the serum after 120 min of tracer synthesis (Additional file 1: Figure S2). This demonstrated minimal radiolysis through defluorination and/or oxidation of the radiotracers by relative lack of new radiochemical peaks [65, 66] at different retention times (R_t_) compared to the original product peak [67, 68].

### Cell uptake studies of ^18^F-KS1

**Fig. 4:**
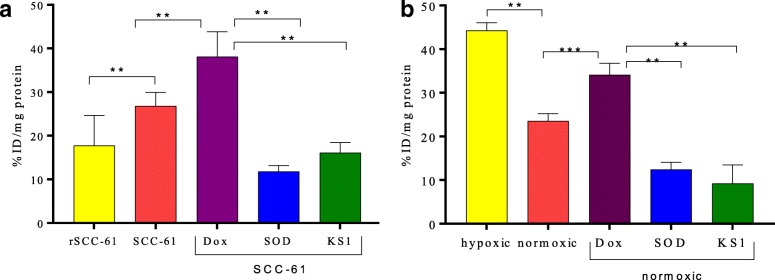
Cell uptake of ^18^F-KS1 at baseline, promoter, and blockade conditions (*n* = 6) after 60 min in vitro in (**a**) SCC-61 and rSCC-61 and (**b**) in PC3 cell line under normoxic and hypoxic conditions after 60 min. The data were expressed as % injected dose (ID)/mg of protein present in each well, with ^**^p ≤ 0.05 considered statistically significant. Plates without ligands are controls

In vitro efficacy of ^18^F-KS1 was studied in various cancer cell lines, with different ROS levels. Among the two matched HNSCC cell lines tested, SCC-61 cells have higher intracellular ROS and protein oxidation compared to rSCC-61 [45, 47]. Radioactive uptake in SCC-61 cells was ~ 1.5-fold higher compared to rSCC-61 uptake (Fig. 4a). Importantly, the uptake was successfully blocked by SOD (~ 60%) and by nonradioactive KS1 (~ 40%). Additionally, treatment with the ROS inducer, doxorubicin, increased the ^18^F-KS1 uptake by ~ 30%. Ascorbate as a blocker lowered the baseline uptake by ~ 48% (%ID/mg protein at baseline 26.81 ± 3.12 vs. with ascorbate = 12.812 ± 1.23). To further test the specificity of ^18^F-KS1 for ROS, radioactive uptake in PCa cells was tested under hypoxic conditions, as several studies have shown that hypoxic conditions promote ROS generation and oxidative stress [69–71]. A reported hypoxia model of PCa cells [72, 73] was tested in which the uptake of ^18^F-KS1 was evaluated in PC3 cells cultured under hypoxic conditions versus normoxic conditions. Radioactive uptake of ^18^F-KS1 in PC3 under hypoxic conditions was ~ twofold higher than the normoxic conditions (Fig. 4b), further indicating the ability of ^18^F-KS1 to measure ROS. Importantly, this uptake was specific and due to ROS because it was blocked by SOD (~ 48%) and nonradioactive KS1 (~ 61%). Furthermore, doxorubicin increased the radioactive uptake by ~ 15%, thus reconfirming ROS specificity. Thus, through these in vitro cell uptake data in different cancer cell lines and manipulations of ROS levels in vitro, we demonstrate excellent binding and specificity of ^18^F-KS1 towards ROS levels in tumor cells.

### MicroPET imaging studies

**Fig. 5:**
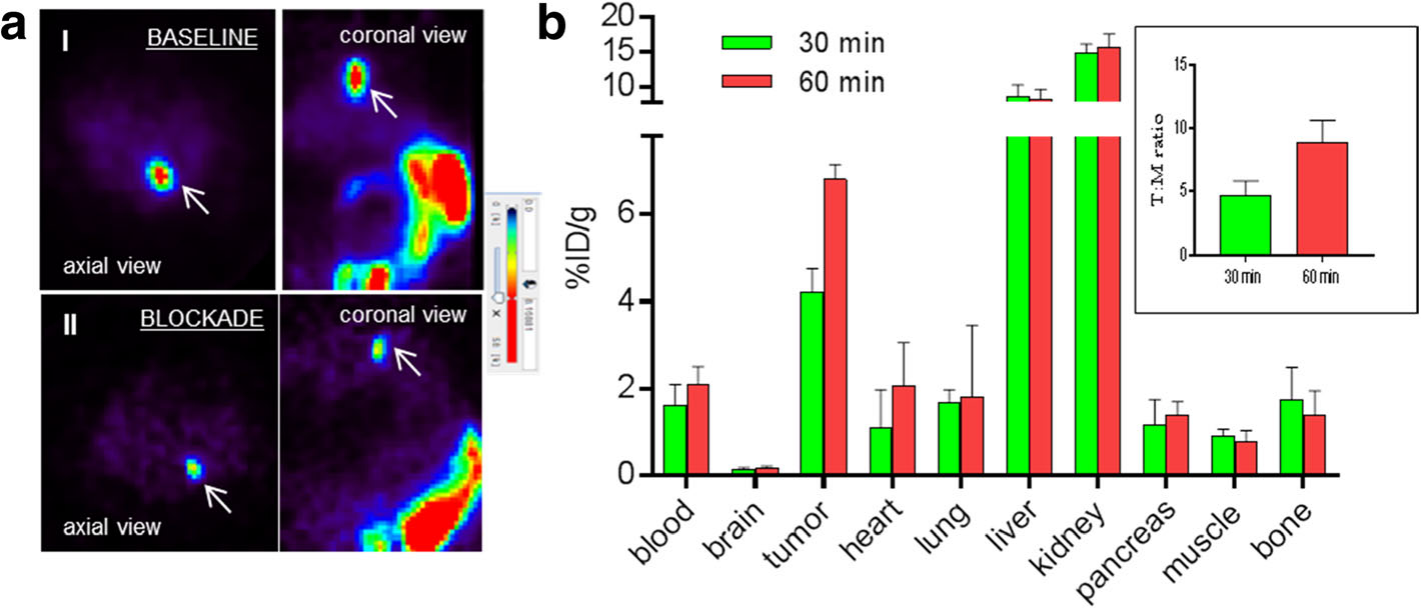
**a** Representative microPET images of ^18^F-KS1 in PC3-bearing mice (*n* = 3): (I) baseline (II) blocking with KS1 in axial and coronal views, with arrow mark highlighting the tumor. **b** Standard biodistribution of ^18^F-KS1 in PC3-bearing mice with values in %ID/g, *p* values ≤ 0.05 considered statistically significant (*n* = 3), with tumor to muscle ratio highlighted inset

To evaluate the first in vivo imaging characteristics of ^18^F-KS1 in a tumor model, microPET imaging studies were performed. Using basic region of interest (ROI) analysis on the microPET scans, ^18^F-KS1 demonstrated (a) high tumor uptake and (b) successful blocking of uptake with nonradioactive KS1 pre-treatment (~ threefold lower than baseline), signifying retained high selectivity and specificity of ^18^F-KS1 in vivo (Fig. 5a).

### In vivo biodistribution

Results of standard biodistribution studies in mice implanted with PCa tumors (*n* = 3/time point) were obtained (Fig. 5b). From 30 to 60 min post-injection, ^18^F-KS1 displayed ~ 1.7-fold increased tumor uptake: %ID/g of 4.23 ± 0.531 (30 min) to 6.81 ± 0.33 (60 min). Bone uptake was lowered from %ID/g of 1.741 ± 0.71 (30 min) to 1.381 ± 0.561 (60 min), suggesting no significant metabolic defluorination in vivo [53]. Additionally, there was > 50% increase in the tumor to muscle (target to nontarget) ratio from 30 min (4.31) to 60 min (8.84) (Fig. 5b inset). Thus, both microPET and biodistribution studies demonstrate high target binding, specificity, and stability of ^18^F-KS1.

## Discussion

The scientific premise of this study is that PET imaging of oxidative stress using a novel ascorbate-based radiotracer will relay critical personalized biochemical information to help design new therapies, individualize existing therapeutic regiments, and better enable clinicians to monitor patient’s therapeutic response in selective applications. Furthermore, the development of our novel translational PET radiotracer may bridge the gap in our understanding of ROS role in tumor progression and therapeutic resistance.

Ascorbate appears to reduce the semistable chromanoxyl radicals through protecting hydrophobic regions of cells, thus generating metabolically active form of lipid antioxidants [74–76]. Emerging pre-clinical and clinical studies led to studies of intravenous ascorbate as a cancer chemotherapeutic agent, as an adjunct to chemotherapy and to ameliorate chemotherapy-induced side effects [77, 78]. Although clinical trials have tested the effects of increasing nutritional supplements of ascorbate in patients with prostate, breast, colorectal, and pancreatic cancer [79], the field has been limited in the ability to detect the downstream products of oxidation manifested as lipid peroxidation and on peripheral products [80–83]. Furthermore, it is unclear as to how ascorbate works with seemingly paradoxical effects in cancer cells; it binds to ROS [84], and it generates ROS at different concentrations [74, 77]. In addition to strategies that aim to decrease ROS, common anticancer chemotherapeutics such as doxorubicin generate ROS for tumor cell death [51, 84–86]. Therefore, the ability to measure ROS levels using ascorbate in tumor cells can significantly advance such therapeutic strategies by enabling (1) personalized regimens based on how oxidative stress contributes to an individual’s disease and (2) ability to monitor real-time effects of any intervention.

The two objectives of our work in this study were to (a) synthesize and characterize a novel ascorbate-based analog and (b) elucidate its initial in vitro and in vivo ROS/RNS selectivity with ascorbate as the reference standard. We designed our structure-activity relationships (SAR) based on the key skeleton of a reported ascorbate derivative [38]. From the lead structure (a potent ascorbate derivative), we retained the 3^1^-enol group for possible ROS reactivities and incorporated substituents at 6^1^ position amenable for [^18^F] radiochemistry. We have synthesized and characterized (*E*)-5-(2-chloroethylidene)-3-((4-(2-fluoroethoxy)benzyl)oxy)-4-hydroxyfuran-2(5H)-one (KS1), as our ascorbate derivative to be studied further for use in ROS detection. We considered the possibility of keto-enol tautomerism in our structure; however, it did not seem to affect the biological activity of the compounds. We measured the in vitro potency of KS1 towards ROS using MitoSOX binding and blocker studies at lower concentrations and compared its activity with ascorbate as the reference standard. Initial preliminary in vitro ROS MitoSOX fluorescence assays demonstrated (a) similar mode of action to that of ascorbate (at lower concentrations) and (b) high ROS binding potency and specificity (demonstrated by SOD blocking) of KS1. SOD blocking specificity suggests that KS1 could be a superoxide peroxide targeting agent [25].

While our initial screening data with MitoSOX is promising, we are currently working on determining our compound’s in vitro efficacy using additional ROS assays [87, 88], with and without commonly used ROS and RNS promoters and blockers [89–95]. The in vitro ROS potency of KS1 was favorable to pursue the next step of PET radiochemistry. We chose a [^18^F]-radiolabeling strategy because of its translational potential with a longer half-life (109.78 min), compared to [^11^C] with a 20-min half-life. ^18^F-KS1 was produced in the TRASIS AIO module, following the standard [^18^F]F^¯^**-**based nucleophilic substitution in high-quality standards (S.A = 100 ± 10 GBq/μmol, radiochemical purity = 95% at EOS). ^18^F-KS1 showed ~ 90% serum stability ex vivo and hence suitable for further in vitro and in vivo investigations. We chose to evaluate the in vitro efficacy of ^18^F-KS1 in two matched cell lines (SCC-61 and rSCC-61) of HNSCC with different ROS levels as well as PCa cell line (PC3) hypoxia model that modulates ROS levels based on oxygen availability. ^18^F-KS1 demonstrated > 2.5-fold ROS selectivity and specificity (in baseline and blocking studies using ROS blockers and promoters). Significant blockade of the baseline uptake with ascorbate implies that ^18^F-KS1 might behave like ascorbate. This suggests that ^18^F-KS1 binding properties including cell trapping and ROS interactions might be similar to ascorbate’s [96, 97]. To build on the in vitro data, we performed small-animal microPET imaging and biodistribution studies of ^18^F-KS1 in mice bearing PCa-PC3 tumors. We initially confirmed ^18^F-KS1’s tumor binding properties in vivo in mice bearing PC3 tumors and found specific binding both at 30 min and 60 min post-injection. While ^18^F-KS1 uptake was still high in blood, kidneys, and liver even after 60 min post-injection, this did not directly affect tumor imaging characteristics of ^18^F-KS1. However, the specificity can be further improved by imaging at later time points, such as 90 and 120 min post-radiotracer injection, especially after further blood pool clearance. To further validate the specificity of ^18^F-KS1 in vivo, we performed a blocking microPET study, where a set of PC3 tumor-bearing mice (*n* = 3) were pre-injected with nonradioactive KS1, before the radiotracer injection. Radioactive uptake was significantly lower (~ threefold) than at baseline, demonstrating the specificity of ^18^F-KS1. Furthermore, we found no immediate adverse reactions of the mice at 15 mg/kg of unlabeled KS1, which is > 100-fold more than the expected radiolabeled dose.

Our initial study with ^18^F-KS1 provides promising data and thus justifies our continuing studies looking at (i) other tumor models in both sexes, (ii) imaging at multiple time points to improve imaging contrast, and (iii) in vivo tracer stability by performing comprehensive metabolite analyses.

## Conclusions

ROS plays a significant role in all stages of cancer growth, including initiation, progression, restaging, and death. ^18^F-KS1, a ROS selective PET ligand, was synthesized and radiolabeled with high radiochemical purity and specific activity. It successfully demonstrated a reliable automated synthesis, a good ex vivo stability, a ROS-specific in vitro profile, and a promising specific tumor uptake in vivo. Biodistribution and microPET imaging studies exhibited good stability, specificity, and tumor uptake in PCa-bearing mice. Thus, we demonstrated initial biological evaluations of a novel PET radiotracer, based on a natural antioxidant with potential to measure ROS levels in a solid tumor in vivo. Based on the data presented here, we hypothesize that our ascorbate-based PET ligand strategy will expand the ascorbate scaffold to measure in vivo oxidative stress in cancer and neurodegenerative and cardiovascular diseases*.* We are therefore pursuing additional studies including mechanistic ROS blocking assays, complete metabolite analyses, and PET imaging studies in other mice models with high oxidative stress.

## Additional file

Additional file 1: **Figure S1.** (A) Representative semiprep HPLC chromatogram with upper UV and lower radio γ trace of ^18^F-KS1 using C18 Phenomenex Luna HPLC column (250 X 10 mm, 10 μA) with 30% acetonitrile in 0.1 M aqueous ammonium formate buffer (pH 6.5) at a flow rate of 5.0 mL/min and UV @ 254 nm.; (B) QC analytical spectrum of ^18^F-KS1 single injection using a C18 Phenomenex Prodigy HPLC column (250 X 4.6 mm, 5 μA) with 45% acetonitrile in 0.1 M aqueous ammonium formate buffer (pH 6.5) at a flow rate of 1.0 mL/min and UV @ 254 nm. UV-mass (top) and radioactive peak (bottom window) were highlighted with arrow marks for the corresponding ^18^F-KS1 product. **Figure S2.** Ex vivo stability of ^18^F-KS1 in human serum sample; radiochemical purity analyzed until 240 min after production (DOCX 210 kb)

## Abbreviations

DHE: Dihydroethidium
EOS: End of synthesis
HNSCC: Head and neck squamous cancer cells
NaOH: Sodium hydroxide
PCa: Prostate cancer
PET: Positron emission tomography
QC-HPLC: Quality control high-performance liquid chromatography
ROS: Reactive oxygen species
