# Supplementary material for: Initial biological evaluations of 18F-KS1, a novel ascorbate derivative to image oxidative stress in cancer
Source: EJNMMI Res. 2019 May 17;9:43. doi: 10.1186/s13550-019-0513-x (PMC6525227; doi:10.1186/s13550-019-0513-x)
Supplement: Supplementary file 1 — Figure S1. (A) Representative semiprep HPLC chromatogram with upper UV and lower radio γ trace of 18F-KS1 using C18 Phenomenex Luna HPLC column (250 X 10 mm, 10 μA) with 30% acetonitrile in 0.1 M aqueous ammonium formate buffer (pH 6.5) at a flow rate of 5.0 mL/min and UV @ 254 nm.; (B) QC analytical spectrum of 18F-KS1 single injection using a C18 Phenomenex Prodigy HPLC column (250 X 4.6 mm, 5 μA) with 45% acetonitrile in 0.1 M aqueous ammonium formate buffer (pH 6.5) at a flow rate of 1.0 mL/min and UV @ 254 nm. UV-mass (top) and radioactive peak (bottom window) were highlighted with arrow marks for the corresponding 18F-KS1 product. Figure S2. Ex vivo stability of 18F-KS1 in human serum sample; radiochemical purity analyzed until 240 min after production (DOCX 210 kb) [file 13550_2019_513_MOESM1_ESM.docx]

**Initial biological evaluations of ^18^F-KS1, a novel ascorbate derivative to image oxidative stress in cancer**


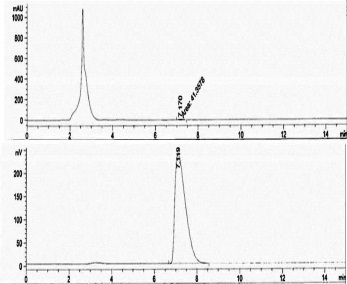


**B.**

**A.**


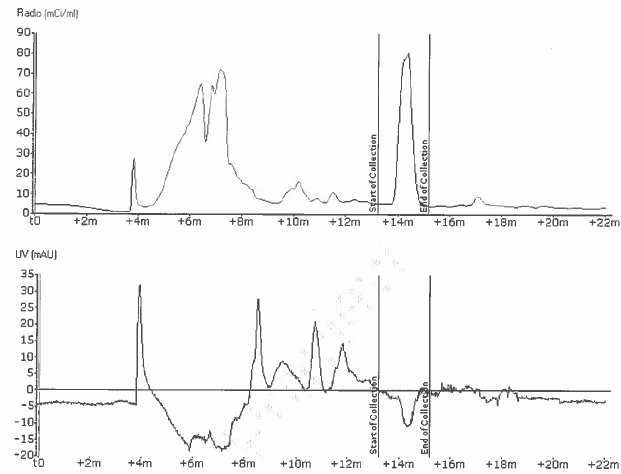

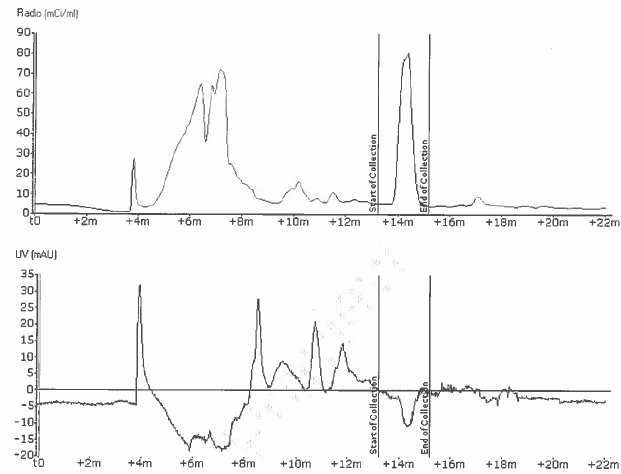


**Figure 1**. (**A**) Representative semiprep HPLC chromatogram with upper UV and lower radio γ trace of ^18^F-**KS1** using C18 Phenomenex Luna HPLC column (250 X 10 mm, 10 µA) with 30% acetonitrile in 0.1M aqueous ammonium formate buffer (pH 6.5) at a flow rate of 5.0 mL/min and UV @ 254 nm.; (**B**) QC analytical spectrum of ^18^F-**KS1** single injection using a C18 Phenomenex Prodigy HPLC column (250 X 4.6 mm, 5 µA) with 45% acetonitrile in 0.1M aqueous ammonium formate buffer (pH 6.5) at a flow rate of 1.0 mL/min and UV @ 254 nm. UV-mass (top) and radioactive peak (bottom window) were highlighted with arrow marks for the corresponding ^18^F-**KS1** product

**Figure 2**: *Ex vivo* stability of ^18^F-**KS1** in human serum sample; radiochemical purity analyzed until 240 min after production
